# Co-Occurrence of Physical and Cognitive Decline in Vervet Monkeys (Chlorocebus aethiops sabaeus)

**DOI:** 10.1093/geroni/igaa057.389

**Published:** 2020-12-16

**Authors:** Brett Frye, Carol Shively, Suzanne Craft, Thomas Register, Matthew Jorgensen, Caitlin Latimer, Christie Scott, Hannah Register

**Affiliations:** 1 Wake Forest School of Medicine, Winston-Salem, North Carolina, United States; 2 Wake Forest University Alzheimer’s Disease Research Center, Winston-Salem, North Carolina, United States; 3 University of Washington, Seattle, Washington, United States

## Abstract

Age-related neurodegeneration associated with Alzheimer’s (AD) disease begins in middle age, well before the onset of symptoms. Therefore, translational models to identify modifiable risk factors in middle-age are needed to understand etiology and identify therapeutic targets. Vervet monkeys (Chlorocebus aethiops sabaeus), like humans, naturally develop several risk factors for AD with age, including obesity, prediabetes, and hypertension. Furthermore, older vervets exhibit accumulation of amyloid and tauopathies, decreased brain volumes, and physical declines in gait speed, suggesting that these NHPs may be useful models of early AD-like neuropathology. Currently, we are investigating the extent to which cognitive and physical decline co-occur in 20 elder (mean age=23 years, ~equivalent to a human age of 80 years) and 10 middle-aged (mean age=11 years) females through assessments of physical performance, executive function, social cognition, and short-term memory. These measures are part of a larger study to integrate physical, social, and cognitive function with measures of body composition, metabolic profiles, CSF, blood, neuroimages, and neuropathology. While tests of social cognition and short-term memory are ongoing, assessments of executive function indicate that performance declines with age (N=26; p<0.05; R-squared=0.23). Furthermore, animals that exhibit slower gait speed also perform poorly on the executive function task (N=26, p<0.05; R-squared=0.25). These preliminary results suggest that accelerated aging co-occurs in multiple systems in vervets. This study will enable examination of temporal relationships between physical and cognitive declines. Ultimately, this comprehensive, integrative whole-body approach will help clarify the mechanisms underlying divergent aging trajectories and inspire interventions that promote multi-system healthy aging.

